# The Pathology and Treatment of Pneumonia

**Published:** 1892-04

**Authors:** J. Clarence Johnson

**Affiliations:** Atlanta, Ga.


ATLANTA
MEDICAL AND SURGICAL JOURNAL.

VOL. IX. APRIL, 1892. NO. 2.

EDITED BY
LUTHER B. GRANDY, M. D., AND MILLER B. HUTCHINS, M. D.,
WITH THE CO-OPERATI ON FO
H. V. M. MILLER, M. D., LL. D., VIRGIL O. HARDON, M. D.,
FLOYD W. McRAE, M. D., AND L. P. KENNEDY, M. D.

### ORIGINAL COMMUNICATIONS.

### THE PATHOLOGY AND TREATMENT OF PNEUMONIA.

By J. CLARENCE JOHNSON, M. D., Atlanta, Ga.

The usual ground upon which a medical treatment claims attention is the disclosure of some new feature of its subject.

Such is not the promise of this paper. Its purpose is simply to discuss a disease of common occurrence, whose pathology is invested with unusual interest and importance because of the peculiar nature and essential function of the parts involved ; and whose successful and scientific treatment merits the most careful study.

The length of the article will not permit us to enter into elaborate detail of many accidental features ; yet we hope to leave untouched no point that might add to a clear and comprehensive consideration of the subject.

Though pneumonia, or more correctly speaking, pneumonitis, has existed, as a distinct disease, almost since the time of Hippocrates, and though it has been a subject of careful observation and research by the ablest minds, its exact nature is still veiled with doubt, and its treatment is yet a matter of discussion. Extended experience with it, as with many other diseases, has given it character and classification without determining the correctness of either.

Notwithstanding the wonderful and useful results of earnest efforts in the various fields of pathology, the limited means at our command do not permit a complete definition of causes and results and the extent of the relation between them. Still, in the light of present knowledge, we are able to reach at least safe and reasonable conclusions.

To determine the true pathology of any disease we must bear in mind the physiological anatomy and functional action of the part or parts it affects. If its course and consequences be not consistent with these, one or the other is false. An inflammation, whatever be its cause, can develop only by a perversion of the vascular action of the part inflamed, and whatever produces the perverted vascular action must do so through the current of blood which the vessels affected convey, or through the intervention of the nerves supplying them. The tissues can resist or eliminate the irritant or poison, and the inflammatory action can be removed only by means of the function whose perversion led to its development.

Anything, then, which disturbs the functional action or the nutrition of a part, or changes the character of the pabulum upon which its function or nutrition depends, is capable of exciting the phenomena which characterize the inflammatory process. Whatever impairs nutrition, or alters the function of a part, does so by altering its blood supply or the action of the nerves governing it. Then a disease depends for its development upon an alteration either of the nervous action or of the blood plasma; and its symptoms will be primary or secondary as the blood or nervous system is first affected.

Accepting these statements as facts, it seems at first an easy matter to understand the causative relation which the various conditions under which pneumonia occurs would bear to it, yet it is this variety of conditions that renders its origin more a matter of doubt.

Until later years it was generally regarded as a simple inflammation of the lungs. By some it is still so considered, but this idea is fast giving place to the opinion that it is a specific fever, due to a micrococcus or bacillus, and that the inflammation is nothing more than a local manifestation of the constitutional disorder.

How far this theory is correct remains to be determined. There is certainly room for doubt as to its correctness. It is in accord with the growing belief that most diseases are produced by germs, and may yet prove the true solution of the question. At present, however, the evidence is not sufficient to convince us, though we would be glad to accept this easy explanation should it be established as a fact.

I do not deny or presume to combat this theory, although it is not in strict accord with my own opinion, but I will use it in comparison for a fair result, if possible.

A cause may often be determined by looking from its effect through its operation.

Let us consider for a moment the occurrence of pneumonia. There is a difference of opinion even as to this. Some hold that it is most frequent at the extremes of life ; others that the converse is true. This discrepancy is, of course, due merely to difference in the experience of the writers, and has no important bearing on the etiology of the disease. All agree that it attacks the laboring class more than others, and especially those whose occupation exposes them to the weather. Vicissitudes of temperature, indeed, is counted an important factor in its production—the measure of its importance remaining a matter of doubt. For this reason pneumonia is most frequent in winter and spring.

It not infrequently occurs in persons with albuminuria, and some stress has been laid upon this as a possible cause. From this, no doubt, arose the idea that pneumonia depends upon some poison in the blood. Upon the same ground malaria is mentioned among the influences that favor or excite its development. These may, as has been proven by facts already stated, afford a fertile soil for its production, and invite invasion by some other poison.

But, inasmuch as these conditions are the exception and not the rule, we may fairly exclude them from the list of causes. In the same way pneumonia may develop in the course of other diseases, as measles and la grippe, but the comparative infrequence with which it follows these proves its occurrence to be accidental rather than incidental to them.

The idea that it is due to a dyscrasia of the blood, whether an inherent cachexia or the result of poison introduced from without, is plausible ; but conflicting with this is the fact that neither scrofula nor tuberculosis predisposes to it. Besides, the suddenness with which it most frequently occurs is not consistent with this idea. It is apparent how an altered blood plasma can affect the functional action and nutrition of the lungs in a way to render them more susceptible to inflammation ; but it is improbable that this alteration could exist to an extent sufficient to produce the inflammation without giving rise to marked or appreciable prodromic symptoms not usual to the disease. Likewise, should it be due to a specific germ or poison, this germ or poison, as would naturally be supposed, from its greater frequency of occurrence in those exposed to atmospheric changes, must enter the circulation directly through the lungs and accumulate in certain quantities before producing the disease, giving rise to essentially the same condition described above. Putting aside these conflicting facts and granting it to be a disease of specific origin, constitutional in character, the germ has not been demonstrated in the blood. Bacilli have been found in the sputa and blood expectorated ; but these same bacilli have been also found in simple bronchitis, which seldom leads, *per se*, to pneumonitis.

Let us go further and compare pneumonia with diseases generally conceded to be of specific origin. The bacillus of typhoid fever has been satisfactorily demonstrated, and it is now supposed to arise from this bacillus and nothing else. So it is with scarlet fever, diphtheria, small-pox and other kindred diseases, which are specific in character, occurring under the same conditions and from the same cause, and never, or rarely, in the same individual twice. They are also self-limited and contagious. Pneumonia does not bear a striking resemblance to either of these in any respect. It is not contagious, and the various conditions under which it occurs prove, at least, that it has many predisposing causes, which, though they may not define, should have weight in determining, its pathology. It is reasonable to suppose that the condition or conditions under which it occurs most frequently bear the most direct causative relation to it. The fact that in most instances it occurs suddenly, like bronchitis and tonsilitis, as the result of exposure, favors the idea that it is more nearly allied to these than to the essential fevers. It may be urged that other diseases, *e. g.*, cholera, known to be of specific origin and constitutional in character, have the same manner of attack as pneumonia; but the evidence is sufficiently strong to convince us that in these it is the nervous system which is primarily and chiefly affected. Be the cause or causes what they may, they differ very little in their results. Pneumonia running a typical course always presents the successive processes of engorgement, red hepatization and grey hepatization, or resolution, though all cases do not present these features in the same degree. In very old people and infants pneumonia is more of a catarrhal nature, and is termed broncho-pneumonia, from the bronchioles being affected.

The first stage, or stage of engorgement, consists of an active determination of blood to and through the lung or lobes involved. This stage usually begins suddenly with a chill, and lasts from a few hours to one or two days. It is characterized by pain in the side at point of irritation, rise of temperature, and more or less cough with expectoration of mucus, the pulse and respiration being accelerated according to the severity of the other symptoms. The crepitant râle may now be heard over seat of developing inflammation. This continuing, the second stage, or red hepatization takes place. The air vesicles are obliterated by the accumulated products of inflammation in the surrounding connective tissue, and more or less effusion into the cavities themselves. This effusion consists of red and white blood corpuscles and serum; a mixture of the red blood corpuscles with the mucus constitutes the brick-dust sputa. This is not always present. In this stage the pain and fever subside, the cough diminishes, the râle, if heard at all, is indistinct, while difficult respiration and debility are the most marked symptoms. This stage varies in duration from two to six days. The third is still more variable. In cases of recovery, it may require four, six, or even ten days. When suppuration takes place, death usually ends the stage in a day or two. If the result is favorable the coloring matter of the red blood corpuscles is first absorbed, leaving the greyish exudation from which the stage gets its name. Following this, the white blood corpuscles or pus cells undergo fatty degeneration and are likewise absorbed. The process continues until the lung is entirely free from the exudation.

Organization of the fibrinous elements sometimes occurs, thickening the connective tissue and lessening the vesicular spaces. This is termed fibrous or chronic pneumonia.

Thus we see that the inflammatory action of pneumonia presents no features different from ordinary inflammation, which cannot be explained by the peculiar character and arrangement of the pulmonary tissue.

Notwithstanding the variability of its course and duration, pneumonia is now classed as a self-limited disease, more, doubtless, for the purpose of being n harmony with the theory that it is an essential fever, than for the appropriateness of the term. Flint says, in his "Practice of Medicine," that it belongs "among those diseases distinguished as self-limited," and yet claims that he has aborted or arrested it by 20-40 grs. of quinine, given at once, or within eight or ten hours.

If it is a self-limited disease, and the fever is essential, it cannot be cut short. If it can be arrested, it is plainly not a self-limited disease.

Scarlet fever, typhoid fever and measles have never been aborted. The experience of those who may read this article will attest the fact that, by timely administration of appropriate remedies, pneumonia may be arrested, or its course very much shortened.

The fact that the majority of cases pass through the successive stages enumerated is due doubtless to the fact that it is seldom seen before red hepatization has begun; which, as a matter of course, must be followed by resolution or absorption, unless death occurs in the meantime. It is true, then, that when the disease has advanced to a certain point it cannot be checked, but must pursue the usual course of any other inflammation; but there is no proof that the first, or stage of engorgement, presupposes the inevitability of the second.

Judging by the testimony of the high authority quoted, the disease may be arrested before solidification takes place. To accomplish this—if it is due to a specific germ—whatever remedy is employed must directly destroy the germ, or quickly eliminate it from the system. The comparative absence in the excretion of any agent supposed to produce the disease is proof conclusive. That relief does not come in this manner; and there is no germicide whose employment seems to have any special influence on the course of the disease. Besides the bacilli found are not dead but living, else, the experiment with regard to their character and action would be valueless.

It is a matter of vital importance to determine which of the theories is correct before entering upon the treatment. If the disease is not self-limited, we should direct our best efforts to checking the inflammation. If it is self-limited such remedies as are usually employed in the treatment of inflammation—and advised and employed even by those who consider it an essential fever—are not only unauthorzed, but unscientific and unsafe.

Regarding it as a constitutional disease, of which the inflammation of the lungs is considered a local manifestation, little is to be done; in fact nothing beyond making the patient comfortable and sustaining the vital powers until the poison has been discharged or has spent its force.

It may be questioned, how far we are justifiable in the use of remedies to combat an essential fever, while we leave the cause unmolested.

Inasmuch as the specific nature of pneumonia has not been determined, and the evidence that it is a local disease, of which the fever is but a symptom, is equally strong, we are justified, both by reason and experience, in treating it as an ordinary inflammation, according to its sthenic, asthenic, or nervous character. Still there remains a question how far we can interfere with the action of the heart without impairing more the functional action of the lobe or lung involved, or lessening the compensatory function of that not affected.

In different periods of the disease different conditions are to be met, and an agent which would be beneficial in one might do harm in another. It is clear that whatever active measures are used to reduce arterial tension should be employed in the beginning of the inflammatory action, or the engorgement that leads to it. Inasmuch as the intensity of the inflammation depends upon the degree of the engorgement, we should attempt to lessen the determination, though we may be ignorant of its cause and the character of the diseases. For this end bleeding was formerly employed.

There are cases in which it may be used with advantage, but we now have efficient substitutes in veratrum and aconite, which are more safe. Either of these will diminish the force and frequency of the pulse, and, if given in time, will do much to check the progress of the inflammation. Veratrum is preferable to aconite, though the latter seems to act better with children.

The best results from either are gotten by giving them at short intervals, in amount sufficient to produce the desired effect, and continuing them in smaller doses, until the indications for their use are removed.

There is some danger in trusting these powerful agents to hands untrained, and it is best for the attending physician himself to administer them at first.

I do not think that the good accomplished by blisters compensates for the irritation and debility they produce. Especially are they contraindicated in young children and those of declining years. Besides, they interfere with further examination of the chest.

Applications of iodine, turpentine or camphor are not only grateful to the feelings of the patient, but do much good. To get the full effect of these applications, they should entirely cover the part affected. Opium is one of our most efficient remedies in the treatment of the first stage. More benefit is to be derived from it at this, than any other period of the disease. Lessening the pain and irritation, as well as restraining the effusion, it directly combats the inflammatory action, and places the tissues in the most favorable condition for recovery.

Its good effects diminish as the disease advances. When hepatization has occurred, it is seldom needed, and in the absence of pain or irritation should not be used. Some claim that it does not retard absorption, but if it affects osmosis one way it will another. If delirium occurs, as is not infrequently the case with those addicted to alcohol, its use may be continued. Otherwise, hyoscyamus or phenacetin is preferable in this stage, and phenacetin is especially indicated when the temperature remains high. Quinine is useful throughout the course of the disease, both for its tonic effect and its influence upon inflammatory action. It may be advantageously combined with the remedies just mentioned, or with opium.

Cough medicines, though usually given, are not always necessary. In many cases the cough is slight, and but little is expectorated. When bronchitis accompanies pneumonia the cough is very troublesome, and something should be done to relieve it. Muriate of ammonia is most universally applicable for this purpose, singly or with other expectorants, as ipecac or wild cherry.

The chief indication for treatment in the second stage is to sustain the vital powers by nutritious food and stimulants, if they are needed. They should be given if the pulse is feeble and rapid and there is general evidence of failing strength.

The third stage requires no special treatment beyond that prescribed for the second. Convalescence may be hastened by attention to the general functions of the body, and the use of tonics or such remedies as the condition of the patient may require.

